# Anhedonia in endometriosis: An unexplored symptom

**DOI:** 10.3389/fpsyg.2022.935349

**Published:** 2022-09-02

**Authors:** Aida Mallorquí, María-Angeles Martínez-Zamora, Francisco Carmona

**Affiliations:** ^1^Clinical Health Psychology Section, Institute of Neuroscience (ICN), Hospital Clínic of Barcelona, Barcelona, Spain; ^2^Department of Gynecology, Clinic Institute of Gynecology, Obstetrics and Neonatology, Hospital Clinic of Barcelona, Faculty of Medicine, University of Barcelona, Institut d´Investigacions Biomèdiques August Pi i Sunyer (IDIBAPS), Barcelona, Spain

**Keywords:** anhedonia, endometriosis, chronic pain, reward system, hedonic tone

## Abstract

Anhedonia is the diminished motivation and sensitivity to pleasurable stimuli. It has been reported to be more prevalent in patients with chronic pain as compared to healthy controls. Endometriosis is a chronic inflammatory systemic disease with a significant psychosocial impact that compromises wellbeing and the day-to-day life of patients. Women with endometriosis show significant psychological distress, even more pervasive when chronic pelvic pain is present. In the current review we will discuss the role of anhedonia in endometriotic chronic pelvic pain. We will also present new lines of research that could lead to more fully clarifying the psychological impact of endometriosis and its detrimental repercussions to quality of life and mental health.

## Introduction

Anhedonia is traditionally defined as the diminished ability to experience pleasure derived from sensory experiences or social interactions. Historically, it has been considered a required symptom for the diagnosis of Depressive Disorders and a residual symptom of Schizophrenia (Berrios and Olivares, 1995; American Psychiatric Association, 2013). However, since its first definition by Ribot in 1897, anhedonia was claimed to be present in “organic situations” as well as in cases of “deep melancholia.” The first scholars interested in anhedonia observed that it could be a temporary state triggered by depression or an enduring trait that characterizes an individual in a stable manner (James, 2003). More recently, some authors have proposed an endophenotype role for anhedonia (Hasler et al., 2004; Pizzagalli et al., 2005; Auerbach et al., 2019), considering it a vulnerability marker present before the onset of depression with the potential to lead to a more pervasive form of the disorder (Shankman et al., 2010).

In 2008, the American Psychiatric Association launched the RDoC (Research Domain Criteria) framework in mental health (Insel et al., 2010). This research framework transitions away from the traditional diagnostic categories to a dimensional approach focused on the analysis of psychological processes and symptoms (Cuthbert, 2014). In this context, anhedonia has been considered in the RdoC framework matrix as a behavioral correlate of the negative valence system, which is the system responsible for responding to aversive situations, such as fear, anxiety, or loss.

Anhedonia is not unitary, contrarily it summarizes multifaceted reward-related deficits with distinctive behavioral and affective manifestations (Franken et al., 2007; Auerbach et al., 2019). Traditionally, it comprised two distinctive aspects: (i) physical anhedonia, which is the blunted hedonic response to physical rewarding stimuli, and (ii) social anhedonia, which is the inability to experience pleasure derived from social interactions (Chapman et al., 1976; Snaith et al., 1995). The Physical Anhedonia Scale and Social Anhedonia Scale were the first psychometric instruments used for measuring anhedonia. Interestingly, some items included in gold-standard depression instruments, such as the Beck Depression Inventory or the Hospital Anxiety and Depression Scales, have also been used to assess anhedonia (Pizzagalli et al., 2005; Langvik et al., 2016).

More recently, anhedonia has been parsed in two distinctive components comprising anticipatory and consummatory aspects of the pleasure experience. Anticipation of pleasure refers to the motivation of the individual toward rewarding stimuli that activate goal-directed behavior (also known as “wanting states” in the neuroscientific literature; Berridge and Kringelbach, 2011). Consummation refers to the liking experience, or the positive emotions elicited by satiation (“liking effects” using Berridge terminology). This dual distinction of the hedonic capacity has been well captured in humans using psychometric measures and neuroimaging techniques (Mallorquí et al., 2014).

**Figure 1:**
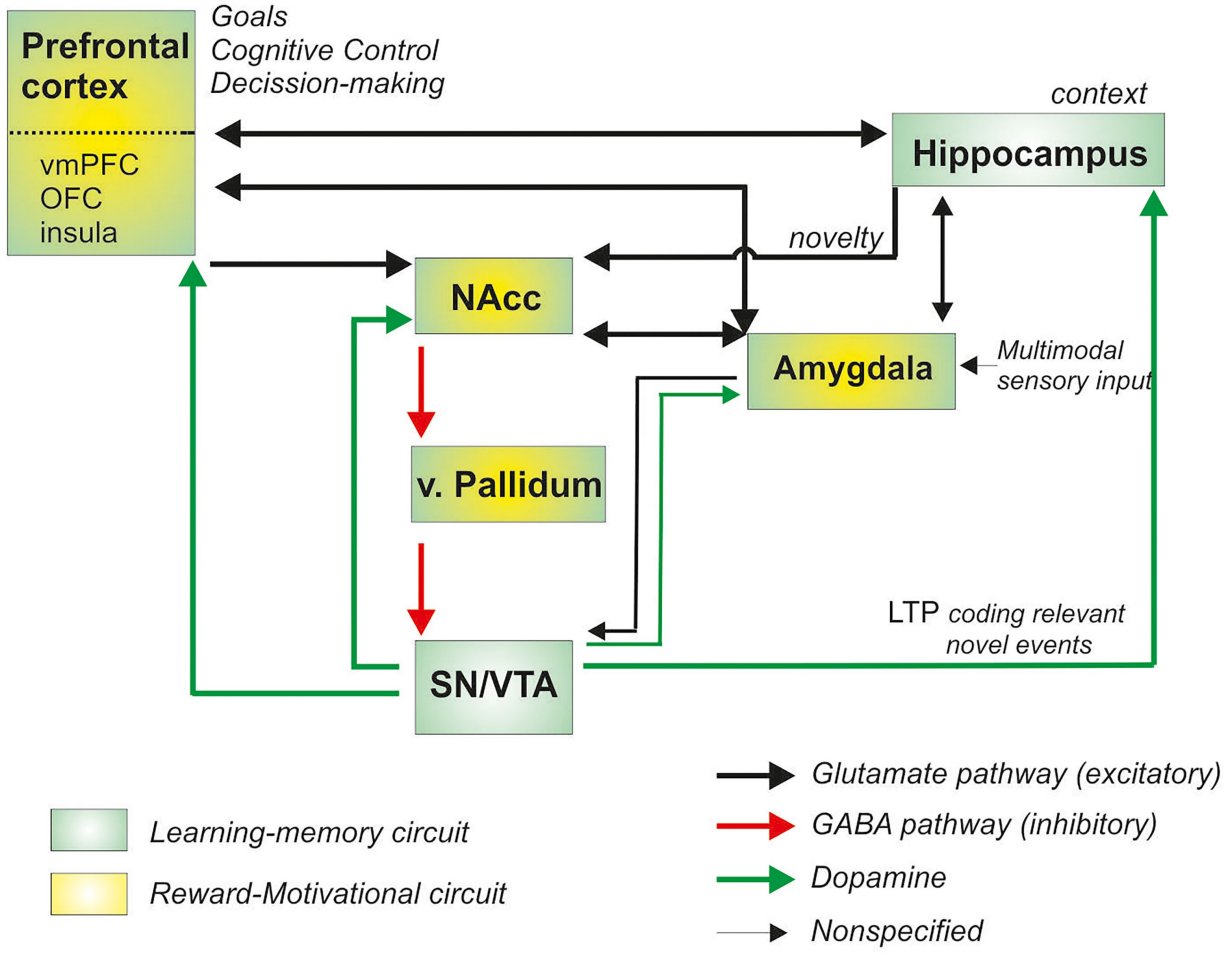
Schematic representation of the brain regions involved in reward processing (adapted and modified from Kelley, 2004; Lisman and Grace, 2005; Camara et al., 2009; Mallorquí et al., 2014; green-yellow boxes; NAcc, Nucleus Accumbens; vmPFC, ventromedial prefrontal cortex; OFC, orbitofrontal cortex; SN Substantia nigra; VTA ventral Tegmental Area; LTP, long-term potentiation; v, ventral).

At the neural level, pleasurable and hedonic experiences recruit an extended neural network of cortico-subcortical structures involved in the regulation of motivational and reward processing. Over the last decade, the use of various neuroimaging techniques has enabled a better understanding of the neural bases at work in the reward system (Wise, 2002; Panksepp, 2004; Robbins and Everitt, 2006; Berridge and Kringelbach, 2008; Camara et al., 2009; Haber and Knutson, 2010), mostly regions in the mesocorticolimbic circuits: the ventral striatum (a core structure in reward processing), the amygdala, the prefrontal cortex (including the orbitofrontal cortex, ventromedial prefrontal cortex or the anterior cingulate cortex), as well as the hippocampal, hypothalamus and insular cortex (Knutson et al., 2001; Yacubian et al., 2006; see for a recent meta-analysis Sescousse et al., 2013). Most importantly, midbrain neurons from the substantia nigra and ventral tegmental area provide dopaminergic input through the mesocortical and mesolimbic pathways to the ventral striatum and prefrontal cortex, regulating the impact and anticipation of hedonic experiences (see Figure 1).

In recent years, anhedonia has gained interest in health beyond psychiatric disorders (Assogna et al., 2011; Treadway and Zald, 2011; Loas, 2014; Mallorquí et al., 2014; Ritsner, 2014a,b). Its transdiagnostic features and presence in non-clinical samples (Harvey et al., 2007) have stimulated new lines of clinical research that highligh the crucial role of anhedonia in chronic health conditions, such as chronic pain. Importantly for the present review, Garland and colleagues (Garland et al., 2020) have recently shown for first time that individuals with different types of chronic pain reported greater levels of anhedonia compared to a well-matched and large sample of healthy controls. More specifically, this association was independent from a clinical diagnosis of Major Depressive Disorder, showing a more complex relationship than the traditional pairing of anhedonia with a subjacent mental health disorder. Subsequently, Carpinelli and colleagues found a significant positive correlation between anhedonia and chronic abdominal pain sufferers, with this relationship not being mediated by depression (Carpinelli et al., 2019). Both studies highlight the scarcity of studies addressing anhedonia in chronic pain and suggest an urgent need for more research in this arena.

Endometriosis is a highly prevalent chronic inflammatory disease where endometrial-like tissue is found outside the uterus, commonly around pelvic areas (Taylor et al., 2021). Rarer cases can present endometrial implants in extrapelvic sites, such as the gastrointestinal and urinary tracts or in more distant areas like the thorax, the central nervous system or even the nasal cavity (Mignemi et al., 2012). Usual symptoms include cyclic or chronic pelvic pain, abnormal uterine bleeding, dyspareunia and infertility. Moreover, quality of life, psychological wellbeing, and mental health are significantly compromised (Facchin et al., 2015; Vitale et al., 2017). In the present review we will address the psychological impact of endometriosis with special attention to chronic pelvic pain and the associated decline of hedonic tone. Finally, we aim to present a new hypothesis on the possible role of anhedonia in endometriosis in order to provide a more fine-grained psychological perspective that could help in developing new lines of intervention.

## Psychosocial impact of endometriosis

Recently recognized as a chronic, systemic inflammatory disease, endometriosis is estimated to affect between 1 in 10 women of reproductive age, globally (Taylor et al., 2021). Pelvic pain and infertility are usually mentioned as common symptoms; however, endometriosis is mostly characterized by a plethora of multifactorial chronic and disabling effects on the body. Symptoms include pelvic pain, dysmenorrhea, non-menstrual pelvic pain, infertility, dyspareunia, dyschezia, dysuria, fatigue, and depression, with a considerable variability in the clinical presentation and level of affectation in patients. Moreover, some severe forms of the disease present alterations of bladder and bowel function with a clear significant reduction in quality of life (Spagnolo et al., 2014).

The pathogenesis of endometriosis is still unknown. Given the substantial comorbidity of endometriosis with autoimmune diseases and the described malfunctioning of many autoimmune cells in women with endometriosis, some authors suggest a possible causal association (Mignemi et al., 2012; Riccio et al., 2018). Thus, a putative abnormal immune response in the peritoneal environment would allow the proliferation of endometrial cells in peripheral sites contributing to the development of the disease (Riccio et al., 2018). In this sense, endometriosis should be considered multifactorial in its etiology, similarly to other autoimmune diseases where emotional stress can act both as a trigger and as a consequence of the disease. This can result in a vicious cycle that ultimately increases the risk of developing a mood disorder (Stojanovich and Marisavljevich, 2008; Benros et al., 2013).

Diagnosis delay and misdiagnosis are one of the problems consistently reported by endometriosis patients (Hudelist et al., 2012; Taylor et al., 2021). The scientific literature is starting to point out to social normalization of dysmenorhea as one of the main reasons behind the average 10-year delay for the diagnosis (Culley et al., 2013; Taylor et al., 2021). From the Health Psychology literature, it has already been demonstrated that normalization in chronic health diseases not only hinders medical diagnosis and treatment, but also has an impact on the person’s cognitive construction of the disease and deters the person from adopting positive health behaviors and appropriate coping strategies. Moreover, when normalization is enacted by close family and friends, it yields unsupportive social interactions that undermine how the person perceives, appraises, and adapts to the disease (Helgeson and Zajdel, 2017).

Beyond its physical symptoms and body systemic impact, endometriosis presents a substantial comorbidity with mental health symptoms and disorders (Laganà et al., 2015, 2017a,b). Currently, depression and anxiety have been the psychological outcomes most often discussed in the endometriosis literature (Delanerolle et al., 2021). Studies consistently report a higher prevalence of depression in women with endometriosis as compared with other groups. As has been reported in a recent meta-analysis, this effect increases significantly when the comparison comprises exclusively well-matched, healthy controls (Gambadauro et al., 2019). Interestingly, in 2021, another systematic review and meta-analysis reported a prevalence of 28.9% for depression and 31.8% for anxiety (Delanerolle et al., 2021).

Besides the symptom-quantitative approach, in 2016, Chen and colleagues (Chen et al., 2016), using the Taiwan National Health Insurance Research Database comprising 10,439 women, reported an increased risk in developing major depressive disorder (hazard ratio: 1.56, 95%, CI:1.24–1.97) and anxiety disorders (hazard ratio: 1.44, 95% CI: 1.22–1.70). Utilizing the same methodology, the same research group also published an increased prevalence of bipolar disorder in patients with endometriosis (Chen et al., 2020). However, neither study presented quantitative data supporting the clinical psychiatric diagnosis. This lack of objective measurement of the symptomatology as well as the neglect of the chronic health context compelled by endometriosis itself has been criticized in the literature (Vitale et al., 2016; Laganà et al., 2017a,b), Further limitations such as the absence of information regarding endometriosis severity, stage of disease and symptom profile compromise obtaining a more nuanced understanding of the relationship between endometriosis and psychiatric disorders (Vitale et al., 2016). Another relevant factor to unravel this relationship is the impact of long-term hormonal contraceptives, which are the first line of medical treatment for endometriosis (Barbara et al., 2021). Despite the general patient satisfaction with treatment and the reported increase in quality of life and emotional wellbeing in women with endometriosis, combined oral contraceptives have been associated with a subsequent use of antidepressants and a first depression diagnosis, with progestin-only products presenting the highest risk (Skovlund et al., 2016).

Endometriosis impacts assumptions about the self and the world held by patients, disrupting perceptions of continuity that are necessary to guide individuals in their everyday lives. Symptoms can affect a female’s sense of identity, education, work plans, sexuality and social functioning among others (Facchin et al., 2017; Vitale et al., 2017). Enhancing different areas of the self, such as self-esteem, body image or self-efficacy, which are necessary to cope with any chronic disease, could have a positive impact in reducing psychological distress (Helgeson and Zajdel, 2017). Notably, in 2017, in a study looking for predictors of psychological distress in endometriosis, Facchin and colleagues (Facchin et al., 2017) demonstrated that patients with a more preserved self, as measured by a compound of variables, showed better mental health outcomes. These results highlight the necessity to apply multimodal treatments to endometriosis and to move away from the biomedical approach. In the same vein in 2020, O’Hara and colleagues. Published the results of a cross-sectional survey of women with endometriosis in the Australian National Action Plan for Endometriosis that highlighted the need to engage with a multidisciplinary team of health practitioners to manage the disease (O’Hara et al., 2020). Consistently, this multidisciplinary approach has been suggested elsewhere in the specialized literature as well (Buggio et al., 2017; Chapron et al., 2019).

However, given the variability in the clinical presentation of symptoms, the deleterious effects of endometriosis on mental health cannot be understood without considering the role of pain and its behavioral consequences. Henceforth, we will focus exclusively on chronic pelvic pain (CPP), a symptom directly related to reward processing.

## Chronic pelvic pain in endometriosis: Psychological distress and anhedonia

Chronic pelvic pain is the most prevalent presentation symptom of endometriosis (Coxon et al., 2018). More than 60% of women with endometriosis report CPP (Maddern et al., 2020). As has been highlighted by some authors, chronic pain can be considered a repeated stressor (Coxon et al., 2018). Interestingly, pain and stress show significant conceptual, physiological and neuroanatomical commonalities. Both are adaptive in protecting the individual from, for example, physical injury or exhaustion; however, when they become chronic, they lead the individual toward long-term physiological and psychological changes that can severely compromise health and quality of life (Abdallah and Geha, 2017). Opioid misuse, abuse and addiction have been consistently associated with chronic pain as well as suicidality. In 2015, a systematic review of 38 studies reported rates of misuse ranging from 21% to 29% and rates of addiction from 8% to 12% (Vowles et al., 2015). Regarding suicide, in 2006, an integrative review of the literature conducted by Tang and Crane reported a lifetime prevalence of suicide attempts that ranged between 5% and 14% and a prevalence of suicidal ideation of 20% among individuals suffering from chronic pain (Tang and Crane, 2006). These data confirm the irrefutable relevance of chronic pain as a global public health concern.

In the case of Endometriosis, when CPP is present, the prevalence of depression can increase up to 86%, demonstrating a steady relationship between both conditions (Lorençatto et al., 2006). Interestingly, some studies point out that CPP, independently from endometriosis, is the main contributor to the mental health and quality of life detriment (Facchin et al., 2015). Sustained inflammation has been proposed as the main mechanism associated with poor mental health (Facchin et al., 2015; Taylor et al., 2021); however, further top-down psychological processes that impact motivation, learning, and decision making could also be significant mediators of this relationship highlighting the need to carry out research in this regard.

Biomedical perspectives of chronic pain have been insufficient to understand the myriad of lifestyle problems that patients encounter (Crombez et al., 2012). Thus, adopting a biopsychosocial approach has become a plausible way to overcome the limitations of the former perspective. From a biopsychosocial view, we recognize that the origins and maintainers of pain are complex and also that a comprehensive understanding requires an integration of psychological and social variables that shape the way in which pain is experienced.

Catastrophizing is a cognitive and emotional coping response to chronic pain that encompasses the tendency to focus on pain symptoms and feelings of helplessness and pessimism (Sullivan et al., 2001; Martin et al., 2011). It is a negative mindset consistently associated with heightened pain experience. In 2011, Martin and colleagues demonstrated in a sample of 115 women with endometriosis that catastrophism predicted a diminished improvement in pain response in endometriosis treatment at 1 year follow-up. Consistently with these results, in 2014, Carey and colleagues showed that catastrophism and young age, were both correlated with persistent pain following specific endometriosis surgery, reinforcing the importance of patients’ beliefs and attitudes held about their pain (Carey et al., 2014).

A full understanding of chronic pain should consider a developmental perspective. A huge body of research conducted with infants and adolescents has demonstrated that the repeated experience of pain at a very young age is a significant factor that increases the odds of suffering chronic pain during the lifetime. Furthermore, the influence of early life experiences, like stress, trauma or peripheral insult has also been reported to influence visceral pain (Fuentes and Christianson, 2018). During adolescence, the central nervous system is highly plastic and long-lasting changes often occur in light of chronic pain. Therefore, suffering acute and cyclic menstrual pain episodes associated with undiagnosed endometriosis could pave the way to chronic pain as a young adult, leading to a challenging life where decisions about education, career or choosing a partner could be constrained.

**Figure 2:**
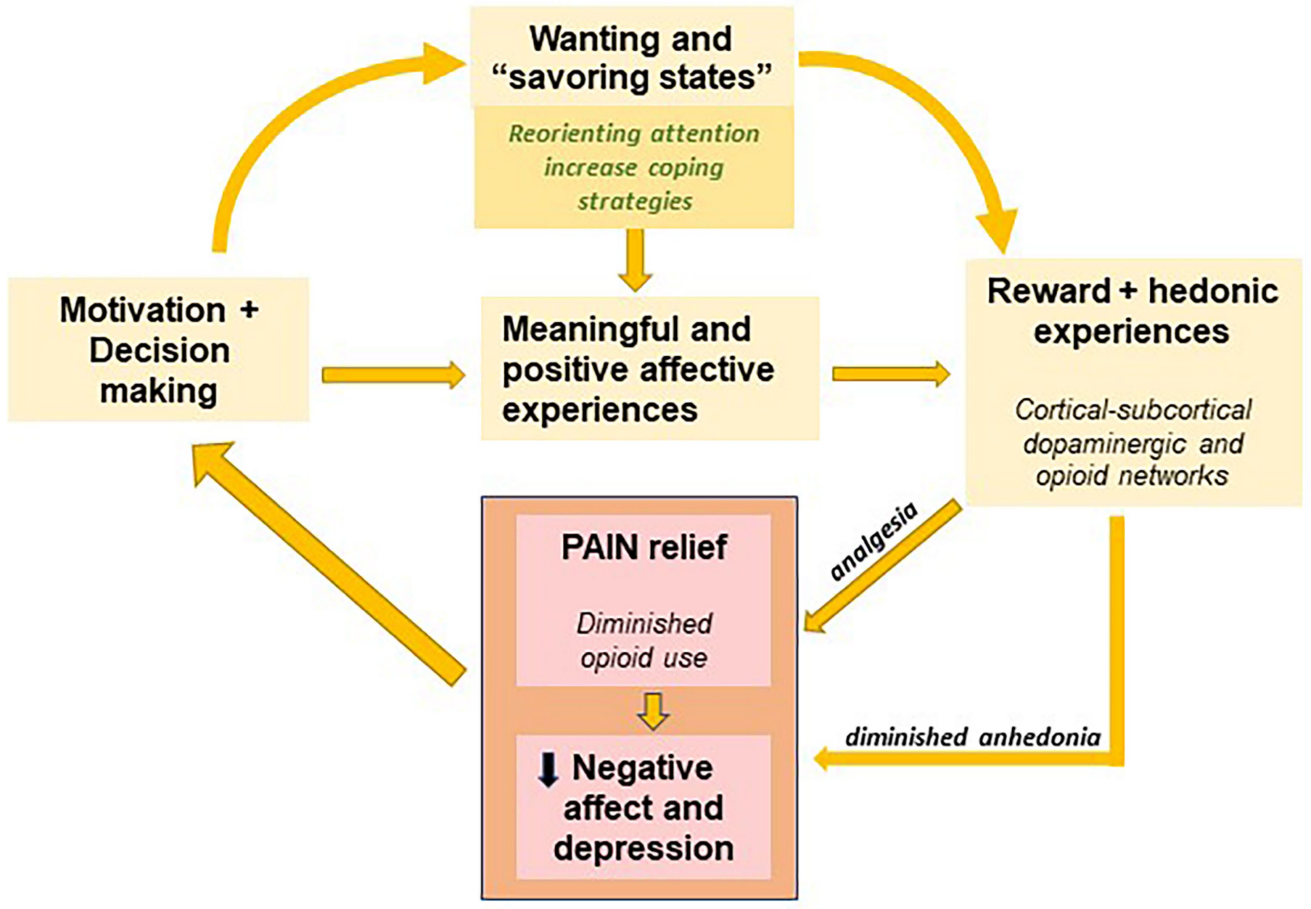
Scheme of the interrelation of the different factors affecting the capacity to experience pleasurable experiences in endometriosis (adapted following the model presented in Garland, 2020). *Yellow boxes* illustrate the motivational processes involved in increasing hedonic and meaningful experiences. The increase in reward and positive hedonic experiences might decrease negative affective states that characterize endometriosis (*pink boxes*), diminishing anhedonia and in turn indirectly potentiating analgesia.

At the present time, no data exist on the capacity to experience pleasure or hedonics in patients suffering endometriosis. In light of the findings discussed in this review and having in mind the high prevalence of chronic pain in endometriosis, the clinical screening of anhedonia in this population seems relevant to help us clarify the mechanisms by which mental and behavioral health could be compromised. Firstly, anhedonia could be playing a role in the elevated rates of depression among endometriosis patients by maintaining depression and negative affective states over time or even leading to more pervasive forms of depression. Secondly, the attention-grabbing quality of pain could contribute in moving attentional resources away from alternative or compensatory hedonic experiences, hindering reward-related learning processes (Garland, 2020). Thirdly, it is already known that pain could result in reward devaluation (Porcelli and Delgado, 2017), affecting the engagement of motivational processes involved in approach and engagement in pleasurable experiences. The complex interaction between these factors, (i) negative attitudes to the illness (catastrophizing), (ii) elevated negative affect, (iii) pain attentional overfocus and (iv) decreased motivation to engage in pleasurable activities, could lead to the progressive and silent development of a blunted hedonic system (see Figure 2).

Therefore, therapeutic interventions for pain release and endometriosis would be more challenging in patients with higher anhedonia, as this condition might prevent patients from exploring and discovering compensatory experiences that might help them defocus from persistent pain. Besides, engaging in rewarding and meaningful activities has been associated with increased analgesia, which could contribute to the amelioration of chronic pain (Thompson et al., 2018; Thorn et al., 2018). Finally, as recent proposals of cognitive treatments for chronic pain are based on the idea of pain defocusing and attention reorienting (i.e., removing the mental contents associated to pain from the spotlight of attention), the chance of engaging in new rewarding and meaningful experiences might promote the reorientation of attention, improving the chances of therapeutic success.

## Discussion

Endometriosis is a chronic systemic disease with a significant psychosocial impact that compromises the wellbeing and day-to-day life of patients. Given its heterogeneity, symptomatic patients should be treated by means of a patient-centered approach with a lifelong management plan (Chapron et al., 2019). Psychological and mental health comorbidities of endometriosis have been identified in the scientific literature. Unfortunately, despite the current amount of psychological data regarding the disease, no model has integrated in a coherent manner all of the correlates found in the literature, and therefore, the resulting psychological perspective is partially fragmented. Clarifying the role of anhedonia in endometriosis could be a helpful step in correcting this, by contributing to design more individualized psychological interventions, clarifying which patient needs them, as well as when and at what doses.

As has been highlighted throughout this review, there are no data available clarifying the role of anhedonia in endometriosis, thus many significant questions remain unanswered. Anhedonia could indeed be a consequence of pain, as it has been observed that chronic pain may alter reward processing by disrupting opioid signaling in the brain (Martikainen et al., 2013). However, further top-down cognitive-behavioral processes involved in goal-setting and decision making (see Figure 1) could also be playing a significant role; CPP could potentially distance the individual from natural and previously acquired resources of pleasure and positive experiences. This could then result in a looping and long lasting deteriorating effect, which continues to worsen symptoms and illness adaptation. Ultimately, the presence of anhedonia in healthy individuals independently from depression should be considered in cases of endometriosis. It is also important to consider its associations with further disease symptoms, such as pelvic organ malfunctioning, which could also disrupt hedonic capacity.

Endometriosis has also an impact in interpersonal aspects such as sexual and social functioning (Vitale et al., 2016). In this regard, the presence of CPP and dyspareunia could be associated with higher levels of social anhedonia leading patients to social avoidance, lack of satisfaction derived from social interactions, and in the specific case of dyspareunia, compromised sexuality, partner intimacy and marital adjustment.

Finally, a preserved hedonic functioning is essential to maintaining wellbeing and directing behavior toward positive experiences. Current scientific literature has demonstrated that women with endometriosis show significant psychological distress, which is even more pervasive when CPP is present. Research that associates chronic pain with anhedonia has been published recently, indicating a significant role of anhedonia in chronic pain, although the relationship is still not fully understood. Therefore, we think that it is imperative to explore anhedonia in endometriosis in order to guide us in designing more precise interventions aimed at optimizing health and wellbeing in women with endometriosis.

## Author contributions

AM and M-AM-Z wrote the paper. FC supervised and revised the last version of the manuscript. All authors contributed to the article and approved the submitted version.

## Conflict of interest

The authors declare that the research was conducted in the absence of any commercial or financial relationships that could be construed as a potential conflict of interest.

## Publisher’s note

All claims expressed in this article are solely those of the authors and do not necessarily represent those of their affiliated organizations, or those of the publisher, the editors and the reviewers. Any product that may be evaluated in this article, or claim that may be made by its manufacturer, is not guaranteed or endorsed by the publisher.

## References

[ref1] Abdallah C. G. Geha P. (2017). Chronic pain and chronic stress: two sides of the same coin? Chronic Stress 1, 247054701770476. doi: 10.1177/2470547017704763, PMID: 28795169 PMC5546756

[ref2] American Psychiatric Association (2013). Diagnostic and Statistical Manual of Mental Disorders. *4th Edn*. Washington, DC: American Psychiatric Association.

[ref3] Assogna F. Cravello L. Caltagirone C. Spalletta G. (2011). Anhedonia in Parkinson’s disease: a systematic review of the literature. Mov. Disord. 26, 1825–1834. doi: 10.1002/MDS.23815, PMID: 21661052

[ref4] Auerbach R. P. Pagliaccio D. Pizzagalli D. A. (2019). Toward an improved understanding of anhedonia. JAMA Psychiatry 76, 571–573. doi: 10.1001/jamapsychiatry.2018.4600, PMID: 30865251 PMC6817369

[ref5] Barbara G. Buggio L. Facchin F. Vercellini P. (2021). Medical treatment for endometriosis: tolerability, quality of life and adherence. Front. Glob. Women's Health 2, 729601. doi: 10.3389/fgwh.2021.729601 34816243 PMC8594049

[ref6] Benros M. E. Waltoft B. L. Nordentoft M. Østergaard S. D. Eaton W. W. Krogh J.. (2013). Autoimmune diseases and severe infections as risk factors for mood disorders: a nationwide study. JAMA Psychiat. 70, 812–820. doi: 10.1001/jamapsychiatry.2013.1111, PMID: 23760347

[ref7] Berridge K. C. Kringelbach M. L. (2008). Affective neuroscience of pleasure: reward in humans and animals. Psychopharmacology 199, 457–480. doi: 10.1007/S00213-008-1099-6, PMID: 18311558 PMC3004012

[ref8] Berridge K. C. Kringelbach M. L. (2011). Building a neuroscience of pleasure and well-being. Psychol. Well-Being 1, 3. doi: 10.1186/2211-1522-1-3, PMID: 22328976 PMC3274778

[ref9] Berrios G. E. Olivares J. M. (1995). The anhedonias: a conceptual history. Hist. Psychiatry 6, 453–470. doi: 10.1177/0957154X9500602403, PMID: 11609003

[ref10] Buggio L. Barbara G. Facchin F. Frattaruolo M. P. Aimi G. Berlanda N. (2017). Self-Management and Psychological-Sexological Interventions in Patients with Endometriosis: Strategies, Outcomes, and Integration into Clinical care. Int. J. Womens Health 9, 281–293. doi: 10.2147/IJWH.S119724, PMID: 28496368 PMC5422563

[ref11] Camara E. Rodriguez-Fornells A. Ye Z. Münte T. F. (2009). Reward networks in the brain as captured by connectivity measures. Front. Neurosci. 3, 350–362. doi: 10.3389/NEURO.01.034.2009/FULL, PMID: 20198152 PMC2796919

[ref12] Carey E. T. Martin C. E. Siedhoff M. T. Bair E. D. As-Sanie S. (2014). Biopsychosocial correlates of persistent postsurgical pain in women with endometriosis. Int. J. Gynecol. Obstet. 124, 169–173. doi: 10.1016/j.ijgo.2013.07.033, PMID: 24290537

[ref13] Carpinelli L. Bucci C. Santonicola A. Zingone F. Ciacci C. Iovino P. (2019). Anhedonia in irritable bowel syndrome and in inflammatory bowel diseases and its relationship with abdominal pain. Neurogastroenterol. Motil. 31, e13531. doi: 10.1111/NMO.13531, PMID: 30628137

[ref14] Chapman L. J. Chapman J. P. Raulin M. L. (1976). Scales for physical and social anhedonia. J. Abnorm. Psychol. 85, 374–382. doi: 10.1037/0021-843X.85.4.374 956504

[ref15] Chapron C. Marcellin L. Borghese B. Santulli P. (2019). Rethinking mechanisms, diagnosis and management of endometriosis. Nat. Rev. Endocrinol. 15, 666–682. doi: 10.1038/s41574-019-0245-z, PMID: 31488888

[ref16] Chen L. C. Hsu J. W. Huang K. L. Bai Y. M. Su T. P. Li C. T.. (2016). Risk of developing major depression and anxiety disorders among women with endometriosis: a longitudinal follow-up study. J. Affect. Disord. 190, 282–285. doi: 10.1016/j.jad.2015.10.030, PMID: 26544610

[ref17] Chen S. Yang Y. Hsu C. Shen Y. C. (2020). Risk of bipolar disorder in patients with endometriosis: a nationwide population-based cohort study. J. Affect. Disord 270, 36–41. doi: 10.1016/j.jad.2020.03.047 32275218

[ref18] Coxon L. Horne A. W. Vincent K. (2018). Pathophysiology of endometriosis-associated pain: a review of pelvic and central nervous system mechanisms. Best Pract. Res. Clin. Obstet. Gynaecol. 51, 53–67. doi: 10.1016/j.bpobgyn.2018.01.014, PMID: 29525437

[ref19] Crombez G. Eccleston C. van Damme S. Vlaeyen J. W. S. Karoly P. (2012). Fear-avoidance model of chronic pain: the next generation. Clin. J. Pain 28, 475–483. doi: 10.1097/AJP.0B013E3182385392 22673479

[ref20] Culley L. Law C. Hudson N. Denny E. Mitchell H. Baumgarten M.. (2013). The social and psychological impact of endometriosis on women’s lives: a critical narrative review. Hum. Reprod. Update 19, 625–639. doi: 10.1093/humupd/dmt027, PMID: 23884896

[ref21] Cuthbert B. N. (2014). The RDoC framework: facilitating transition from ICD/DSM to dimensional approaches that integrate neuroscience and psychopathology. World Psychiatry 13, 28–35. doi: 10.1002/WPS.20087, PMID: 24497240 PMC3918011

[ref22] Delanerolle G. Ramakrishnan R. Hapangama D. Zeng Y. Shetty A. Elneil S.. (2021). A systematic review and meta-analysis of the endometriosis and mental-health Sequelae; the ELEMI project. Women’s Health 17, 174550652110197. doi: 10.1177/17455065211019717, PMID: 34053382 PMC8182632

[ref23] Facchin F. Barbara G. Dridi D. Alberico D. Buggio L. Somigliana E.. (2017). Mental health in women with endometriosis: searching for predictors of psychological distress. Hum. Reprod. 32, 1855–1861. doi: 10.1093/humrep/dex249, PMID: 28854724

[ref24] Facchin F. Barbara G. Saita E. Mosconi P. Roberto A. Fedele L.. (2015). Impact of endometriosis on quality of life and mental health: pelvic pain makes the difference. J. Psychosom. Obstet. Gynecol. 36, 135–141. doi: 10.3109/0167482X.2015.1074173, PMID: 26328618

[ref25] Franken I. H. A. Rassin E. Muris P. (2007). The assessment of anhedonia in clinical and non-clinical populations: further validation of the Snaith–Hamilton pleasure scale (SHAPS). J. Affect. Disord. 99, 83–89. doi: 10.1016/J.JAD.2006.08.020, PMID: 16996138

[ref26] Fuentes I. M. Christianson J. A. (2018). The influence of early life experience on visceral pain. Front. Syst. Neurosci. 12. doi: 10.3389/FNSYS.2018.00002/FULL, PMID: 29434541 PMC5790786

[ref27] Gambadauro P. Carli V. Hadlaczky G. (2019). Depressive symptoms among women with endometriosis: a systematic review and meta-analysis. Am. J. Obstet. Gynecol. 220, 230–241. doi: 10.1016/j.ajog.2018.11.123, PMID: 30419199

[ref28] Garland E. L. (2020). Psychosocial intervention and the reward system in pain and opioid misuse: new opportunities and directions. Pain 161, 2659–2666. doi: 10.1097/j.pain.0000000000001988, PMID: 33197164 PMC7678811

[ref29] Garland E. L. Trostheim M. Eikemo M. Ernst G. Leknes S. (2020). Anhedonia in chronic pain and prescription opioid misuse. Psychol. Med. 50, 1977–1988. doi: 10.1017/S0033291719002010, PMID: 31422776

[ref30] Haber S. N. Knutson B. (2010). The reward circuit: linking primate anatomy and human imaging. Neuropsychopharmacology 35, 4–26. doi: 10.1038/npp.2009.129, PMID: 19812543 PMC3055449

[ref31] Harvey P. O. Pruessner J. Czechowska Y. Lepage M. (2007). Individual differences in trait anhedonia: a structural and functional magnetic resonance imaging study in non-clinical subjects. Mol. Psychiatry 12, 767–775. doi: 10.1038/sj.mp.4002021, PMID: 17505465

[ref32] Hasler G. Drevets W. C. Manji H. K. Charney D. S. (2004). Discovering Endophenotypes for major depression. Neuropsychopharmacology 29, 1765–1781. doi: 10.1038/sj.npp.1300506, PMID: 15213704

[ref33] Helgeson V. S. Zajdel M. (2017). Adjusting to chronic health conditions. Annu. Rev. Psychol. 68, 545–571. doi: 10.1146/ANNUREV-PSYCH-010416-044014 28051935

[ref34] Hudelist G. Fritzer N. Thomas A. Niehues C. Oppelt P. Haas D.. (2012). Diagnostic delay for endometriosis in Austria and Germany: causes and possible consequences. Hum. Reprod. 27, 3412–3416. doi: 10.1093/humrep/des316, PMID: 22990516

[ref35] Insel T. Cuthbert B. Garvey M. Heinssen R. Pine D. S. Quinn K.. (2010). Research domain criteria (RDoC): toward a new classification framework for research on mental disorders. Am. J. Psychiatr. 167, 748–751. doi: 10.1176/APPI.AJP.2010.09091379, PMID: 20595427

[ref36] James W. (2003). *The Varieties of Religious Experience*. doi: 10.4324/9780203398470/VARIETIES-RELIGIOUS-EXPERIENCE-WILLIAM-JAMES

[ref37] Kelley A. E. (2004). Memory and addiction: shared neural circuitry and molecular mechanisms. Neuron 44, 161–179. 15450168 10.1016/j.neuron.2004.09.016

[ref38] Knutson B. Adams C. M. Fong G. W. Hommer D. Varner J. Kerich M.. (2001). Anticipation of increasing monetary reward selectively recruits nucleus accumbens. J. Neurosci. 21, –RC159. doi: 10.1523/JNEUROSCI.21-16-J0002.2001, PMID: 11459880 PMC6763187

[ref41] Laganà A. S. Condemi I. Retto G. Muscatello M. R. A. Bruno A. Zoccali R. A.. (2015). Analysis of psychopathological comorbidity behind the common symptoms and signs of endometriosis. Eur. J. Obstet. Gynecol. Reprod. Biol. 194, 30–33. doi: 10.1016/J.EJOGRB.2015.08.015, PMID: 26319653

[ref40] Laganà A. S. La Rosa V. Petrosino B. Vitale S. G. (2017a). Comment on “risk of developing major depression and anxiety disorders among women with endometriosis: a longitudinal follow-up study”. J. Affect. Disord. 208, 673–672. doi: 10.1016/j.jad.2016.07.016 27451810

[ref42] Laganà A. S. la Rosa V. L. Rapisarda A. M. C. Valenti G. Sapia F. Chiofalo B.. (2017b). Anxiety and depression in patients with endometriosis: impact and management challenges. Int. J. Women's Health 9, 323–330. doi: 10.2147/IJWH.S119729, PMID: 28553145 PMC5440042

[ref43] Langvik E. Hjemdal O. Nordahl H. M. (2016). Personality traits, gender differences and symptoms of anhedonia: what does the hospital anxiety and depression scale (HADS) measure in nonclinical settings? Scand. J. Psychol. 57, 144–151. doi: 10.1111/sjop.12272, PMID: 26861735

[ref300] Lisman J. E. Grace A. A. (2005). The hippocampal-VTA loop: controlling the entry of information into long-term memory. Neuron 46, 703–713. 15924857 10.1016/j.neuron.2005.05.002

[ref44] Loas G. (2014). “Anhedonia and risk of suicide: an overview,” in Anhedonia: A Comprehensive Handbook. Vol. II. ed. M. S. Ritsner (New York: Springer), 247–253.

[ref45] Lorençatto C. Petta C. A. Navarro M. J. Bahamondes L. Matos A. (2006). Depression in women with endometriosis with and without chronic pelvic pain. Acta Obstet. Gynecol. Scand. 85, 88–92. doi: 10.1080/00016340500456118 16521687

[ref46] Maddern J. Grundy L. Castro J. Brierley S. M. (2020). Pain in endometriosis. Front. Cell. Neurosci. 14. doi: 10.3389/FNCEL.2020.590823/FULL, PMID: 33132854 PMC7573391

[ref47] Mallorquí A. Padrao G. Rodriguez-Fornells A. (2014). “Electrophysiological signatures of reward processing in anhedonia,” in Anhedonia: A Comprehensive Handbook. Conceptual Issues and Neurobiological Advances. Vol. I. ed. M. S. Ritsner (New York: Springer), 245–278.

[ref48] Martikainen I. K. Peciña M. Love T. M. Nuechterlein E. B. Cummiford C. M. Green C. R.. (2013). Alterations in endogenous opioid functional measures in chronic back pain. J. Neurosci. 33, 14729–14737. doi: 10.1523/JNEUROSCI.1400-13.2013, PMID: 24027273 PMC3771036

[ref49] Martin C. E. Johnson E. Wechter M. E. Leserman J. Zolnoun D. A. (2011). Catastrophizing: a predictor of persistent pain among women with endometriosis at 1 year. Hum. Reprod. 26, 3078–3084. doi: 10.1093/humrep/der292, PMID: 21900393 PMC3196877

[ref51] Mignemi G. Facchini C. Raimondo D. Montanari G. Ferrini G. Seracchioli R. (2012). A case report of nasal endometriosis in a patient affected by Behcet’s disease. J. Minim. Invasive Gynecol. 19, 514–516. doi: 10.1016/j.jmig.2012.03.005, PMID: 22748956

[ref52] Neuron A. K. (2004). Memory and addiction: shared neural circuitry and molecular mechanisms. Neuron 44, 161–179. doi: 10.1016/j.neuron.2004.09.016 15450168

[ref53] O’Hara R. Rowe H. Fisher J. (2020). Managing endometriosis: a cross-sectional survey of women in Australia. J. Psychosom. Obstet. Gynaecol. 1–8. doi: 10.1080/0167482X.2020.1825374 [Epub ahead of print]. 33050751

[ref55] Panksepp J. (2004). *Affective neuroscience: The foundations of human and animal emotions*. Available at: https://books.google.es/books?hl=ca&lr=&id=qqcRGagyEuAC&oi=fnd&pg=PR13&dq=panksepp+the+foundations+of+human+and+animal+emotions&ots=-PK5XYykrB&sig=gjSgUJ8b4yrmpVY9F_dJYJr67UQ

[ref56] Pizzagalli D. A. Jahn A. L. O’Shea J. P. (2005). Toward an objective characterization of an anhedonic phenotype: a signal-detection approach. Biol. Psychiatry 57, 319–327. doi: 10.1016/J.BIOPSYCH.2004.11.026, PMID: 15705346 PMC2447922

[ref57] Porcelli A. Delgado M. R. (2017). Stress and decision making: effects on valuation, learning, and risk-taking. Curr. Opin. Behav. Sci. 14, 33–39. doi: 10.1016/j.cobeha.2016.11.015 28044144 PMC5201132

[ref58] Riccio L. Santulli P. Marcellin L. Abrão M. S. Batteux F. Chapron C. (2018). Immunology of endometriosis. Best Pract. Res. Clin. Obstet. Gynaecol. 50, 39–49. doi: 10.1016/j.bpobgyn.2018.01.010 29506962

[ref59] Ritsner M. S. (2014a). “Anhedonia: a comprehensive handbook volume I: conceptual issues and neurobiological advances,” in Anhedonia: A Comprehensive Handbook Volume I: Conceptual Issues and Neurobiological Advances. New York: Springer, 1–352.

[ref60] Ritsner M. S. (2014b). “Anhedonia: a comprehensive handbook volume II: neuropsychiatric and physical disorders,” in Anhedonia: A Comprehensive Handbook Volume II: Neuropsychiatric and Physical Disorders. New York: Springer, 1–328.

[ref61] Robbins T. W. Everitt B. J. (2006). A role for mesencephalic dopamine in activation: commentary on Berridge. Psychopharmacology 191, 433–437. doi: 10.1007/S00213-006-0528-7 16977476

[ref63] Sescousse G. Caldú X. Segura B. Dreher J. C. (2013). Processing of primary and secondary rewards: a quantitative meta-analysis and review of human functional neuroimaging studies. Neurosci. Biobehav. Rev. 37, 681–696. doi: 10.1016/J.NEUBIOREV.2013.02.002, PMID: 23415703

[ref64] Shankman S. A. Nelson B. D. Harrow M. Faull R. (2010). Does physical anhedonia play a role in depression? A 20-year longitudinal study. J. Affect. Disord. 120, 170–176. doi: 10.1016/j.jad.2009.05.002, PMID: 19467713 PMC2794988

[ref65] Skovlund C. W. Mørch L. S. Kessing L. V. Lidegaard Ø. (2016). Association of hormonal contraception with depression. JAMA Psychiat. 73, 1154–1162. doi: 10.1001/jamapsychiatry.2016.2387, PMID: 27680324

[ref66] Snaith R. P. Hamilton M. Morley S. Humayan A. Hargreaves D. Trigwell P. (1995). A scale for the assessment of hedonic tone the Snaith–Hamilton pleasure scale. Br. J. Psychiatry 167, 99–103. doi: 10.1192/BJP.167.1.99, PMID: 7551619

[ref67] Spagnolo E. Zannoni L. Raimondo D. Ferrini G. Mabrouk M. Benfenati A.. (2014). Urodynamic evaluation and anorectal manometry pre-and post-operative bowel shaving surgical procedure for posterior deep infiltrating endometriosis: a pilot study. J. Minim. Invasive Gynecol. 21, 1080–1085. doi: 10.1016/j.jmig.2014.05.012, PMID: 25544711

[ref68] Stojanovich L. Marisavljevich D. (2008). Stress as a trigger of autoimmune disease. Autoimmun. Rev. 7, 209–213. doi: 10.1016/j.autrev.2007.11.007 18190880

[ref69] Sullivan M. Thorn B. Haythornthwaite J. A. Keefe F. Martin M. Bradley L. A.. (2001). Theoretical perspectives on the relation between catastrophizing and pain. Clin. J. Pain 17, 52–64. doi: 10.1097/00002508-200103000-00008 11289089

[ref70] Tang N. K. Y. Crane C. (2006). Suicidality in chronic pain: a review of the prevalence, risk factors and psychological links. Psychol. Med. 36, 575–586. doi: 10.1017/S0033291705006859, PMID: 16420727

[ref71] Taylor H. S. Kotlyar A. M. Flores V. A. (2021). Endometriosis is a chronic systemic disease: clinical challenges and novel innovations. Lancet 397, 839–852. doi: 10.1016/S0140-6736(21)00389-5, PMID: 33640070

[ref72] Thompson S. J. Pitcher M. H. Stone L. S. Tarum F. Niu G. Chen X.. (2018). Chronic neuropathic pain reduces opioid receptor availability with associated anhedonia in rat. Pain 159, 1856–1866. doi: 10.1097/J.PAIN.0000000000001282, PMID: 29794614 PMC6095806

[ref73] Thorn B. E. Eyer J. C. Morais C. A. van Dyke B. P. Torres C. A. Burns J. W.. (2018). Literacy-adapted cognitive behavioral therapy versus education for chronic pain at low-income clinics: a randomized controlled trial. Acpjournals.Org 168, 471–480. doi: 10.7326/M17-0972, PMID: 29482213

[ref74] Treadway M. T. Zald D. H. (2011). Reconsidering anhedonia in depression: lessons from translational neuroscience. Neurosci. Biobehav. Rev. 35, 537–555. doi: 10.1016/J.NEUBIOREV.2010.06.006, PMID: 20603146 PMC3005986

[ref75] Vitale S. G. Petrosino B. La Rosa V. L. Rapisarda A. M. Laganà A. S. (2016). A systematic review of the association between psychiatric disturbances and endometriosis. J. Obstet. Gynaecol. Can 38, 1079–1080. doi: 10.1016/j.jogc.2016.09.008, PMID: 27986180

[ref76] Vitale S. G. La Rosa V. L. Rapisarda A. M. C. Lagana A. S. (2017). Impact of endometriosis on quality of life and psychological well-being. J. Psychosom. Obstet. Gynecol. 38, 317–319. doi: 10.1080/0167482X.2016.1244185 27750472

[ref77] Vowles K. E. McEntee M. L. Julnes P. S. Frohe T. Ney J. P. van der Goes D. N. (2015). Rates of opioid misuse, abuse, and addiction in chronic pain: a systematic review and data synthesis. Pain 156, 569–576. doi: 10.1097/01.j.pain.0000460357.01998.f1, PMID: 25785523

[ref78] Wise R. A. (2002). Brain reward circuitry: insights from Unsensed incentives. Neuron 36, 229–240. doi: 10.1016/S0896-6273(02)00965-0 12383779

[ref79] Yacubian J. Gläscher J. Schroeder K. Sommer T. Braus D. F. Büchel C. (2006). Dissociable systems for gain-and loss-related value predictions and errors of prediction in the human brain. J. Neurosci. 26, 9530–9537. doi: 10.1523/JNEUROSCI.2915-06.2006, PMID: 16971537 PMC6674602

